# Prospective Genomic Surveillance of Severe Febrile Illness in Tanzanian Children Identifies High Mortality and Resistance to First-Line Antibiotics in Bloodstream Infections

**DOI:** 10.4269/ajtmh.25-0522

**Published:** 2026-02-26

**Authors:** Teresa B. Kortz, Victoria T. Chu, Raya Y. Mussa, Erasto M. Sunzula, Victoria M. Mlele, Cecilia L. Msemwa, Lushona K. Mathias, Namala P. Mkopi, Ryan J. Ward, Kathleen S. Sun, Jazmin M. Baez Maidana, Anneka M. Hooft, Juma A. Mfinanga, Joseph L. DeRisi, Hendry R. Sawe, Joel P. Manyahi, Charles R. Langelier

**Affiliations:** ^1^ University of California, San Francisco, California;; ^2^ University of California San Francisco, Institute for Global Health Sciences, San Francisco, California;; ^3^ Muhimbili National Hospital, Dar es Salaam, Tanzania;; ^4^ Muhimbili University of Health and Allied Sciences, Dar es Salaam, Tanzania;; ^5^ Chan Zuckerberg Biohub, San Francisco, California

## Abstract

Severe febrile illness (SFI), a major cause of child mortality in Sub-Saharan Africa (SSA), is frequently caused by bloodstream infections (BSI), increasingly caused by antimicrobial-resistant organisms. However, antimicrobial resistance (AMR) prevalence and patterns in this population are largely unknown. We conducted a prospective cohort study of children aged 28 days to 14 years with SFI at a referral hospital in Tanzania (July 2022–September 2023) to determine the prevalence, pathogen profile, and AMR patterns of BSIs. Blood cultures were performed on all participants. Isolates underwent AMR testing and whole genome sequencing (WGS) to identify AMR genes and assess genetic relatedness. The association between BSI and mortality was assessed using logistic regression. Among the 392 enrolled children (median age 17.8 months), 5.2% (*n* = 20) had culture-confirmed BSI, with a case fatality rate of 45%. Gram-negative bacteria predominated (*Escherichia coli, Klebsiella pneumoniae*), with 79% of isolates exhibiting phenotypic resistance to ceftriaxone, a first-line antimicrobial. WGS revealed extended–spectrum β-lactamase genes (*CTX-M-15*, *CTX-M-27*) in most Enterobacterales isolates and identical isolates among 2/4 patients with *Streptococcus pneumoniae* and 2/3 *Candida albicans* infections. Children with BSI had significantly higher mortality than those without BSI (adjusted odds ratio 3.9; 95% CI: 1.5–10.1, *P* <0.01). These findings emphasize the urgent need for expanded AMR surveillance systems, empiric antibiotic regimens tailored to local AMR patterns, and culture-independent diagnostics in SSA. These data are critical for understanding the cause of severe infection and have the potential to guide policy aimed at reducing child mortality from BSI.

## INTRODUCTION

Severe febrile illness (SFI) is a major cause of childhood death in Sub-Saharan Africa (SSA).^1^^,^^2^ Among children hospitalized for SFI, bloodstream infections (BSIs) carry particularly high mortality,^3^ and, despite global efforts, BSI prevalence has not decreased over recent decades.^4^ BSI treatment is increasingly complicated by antimicrobial resistance (AMR),^4^^–^^6^ a major global public health threat associated with 4.7 million global deaths in 2021.^7^ The SSA region has the highest number of AMR-associated deaths, accounting for 20% of the global burden^7^; an estimated 59% of these deaths occurred in children.^7^ However, AMR prevalence in children hospitalized for SFI is largely unknown in SSA.

Reducing the burden of AMR in SSA is hindered by a lack of data on prevalence, phenotypic resistance patterns, and genomic features of resistant microbes, especially among children. Although empiric antibiotic treatment is frequently recommended for SFIs given the potential for bacterial BSI, including invasive nontyphoid *Salmonella*, in many SSA countries, it is unknown whether empiric antibiotic therapy is appropriate to treat local BSI–causing pathogens. Moreover, recent studies show an alarming increase in first-line antimicrobial resistance, emphasizing the urgent need to characterize AMR patterns in depth.^3^^,^^8^

Even less is known about the genomic features of pediatric community-acquired BSI pathogens and associated antimicrobial resistance genes (ARGs). To address these gaps, we carried out a prospective cohort study in Tanzanian children with SFI and assessed the prevalence and clinical features of BSI. Using whole genome sequencing (WGS), we identified causative BSI pathogens and ARGs and tested for associations with mortality.

## MATERIALS AND METHODS

### Study design and participants.

We screened children presenting to the Emergency Medicine Department (EMD) at Muhimbili National Hospital (MNH), the national referral hospital for Tanzania (July 26, 2022–September 20, 2023). Inclusion criteria were children (ages ≥28 days to ≤14 years) with SFI, defined as fever (axillary temperature >38°C or history of fever) and ≥1 World Health Organization warning sign(s): respiratory distress, altered mental status, impaired perfusion, or at-risk for dehydration (operational definitions in Supplemental Table 1).^9^ We excluded children who had acute trauma or burns; fever not due to infection (e.g., rheumatologic disease); known epilepsy with altered mental status solely due to convulsion; those presenting with cardiac arrest; those weighing <4 kg; and those whose guardians were not available or declined participation. MNH cares for a disproportionate number of children with malignancy and congenital heart disease; therefore, children with a diagnosis consistent with either condition were excluded. The primary outcome was in-hospital mortality; the secondary outcome was prevalence of BSI (e.g., blood culture positive for a likely bacterial/fungal pathogen). The sample size was determined a priori to detect an absolute difference in mortality of 20% between participants with BSI versus those without BSI, assuming a 6% prevalence of BSI. We followed the Strengthening the Reporting of Observational studies in Epidemiology (STROBE) reporting guidelines (Supplemental Data 1).^10^

The study was approved by the Muhimbili University of Health and Allied Sciences (MUHAS) (DA.282/298/01.C/374), Tanzanian National Institute for Medical Research (NIMR) (NIMR/HQ/R.8a/Vol. IX/3576), and University of California, San Francisco (19–27627) Institutional Review Boards. We obtained written informed consent from guardians and verbal participant assent when appropriate. Participants received routine standard of care, including antibiotics, intravenous fluids, and antimalarial medications as needed. Children living with HIV were continued on antiretrovirals.

### Laboratory procedures.

Blood was collected within 4 hours of presentation via venipuncture by using sterile technique. Point-of-care testing included malaria screening (SD bioline-Pf/*Pan*), HIV screening (HIV-1/2 Alere), blood sugar, and hemoglobin. For blood culture, 1.5–3.0 mL of blood were collected in BD BACTEC Peds Plus/F culture vials (Becton Dickinson, Franklin Lakes, NJ). All samples were immediately transported to the MUHAS Bacteriological Research Laboratory at room temperature for processing.

Blood culturing was performed using a BD BACTEC FX40 (Becton Dickinson) according to the manufacturer’s instructions. Positive cultures underwent Gram stain and subculturing onto the following plates: sheep blood agar, chocolate agar, and MacConkey agar. Blood agar and chocolate agar plates were incubated at 35°C with 5–10% carbon dioxide, and MacConkey agar plates were incubated at 37°C. All plates were incubated for 18–24 hours.

We used API20E (BioMérieux, Marcy, I’Etoile, France) and Staphaurex (Remel Europe Ltd, Dartford, United Kingdom) to identify *Enterobacterales* and *Staphylococcus aureus*, respectively. Antimicrobial susceptibility was determined by the Kirby–Bauer disk diffusion method by following international standards.^11^ We confirmed extended–spectrum β-lactamase (ESBL) production by double disk approximation methods for *Enterobacterales* resistant to ceftazidime/ceftriaxone. The study microbiologist (JM) performed quality control and results confirmation. Contaminants were identified biochemically and genomically. The following organisms were considered contaminants and excluded from analysis: coagulase-negative *Staphylococcus* spp., *Corynebacterium* spp., *Micrococcus* spp., *Bacillus* spp., and *Brevibacterium* spp.

For WGS, DNA was extracted from isolates (Zymo Quick-DNA Fungal/Bacterial Miniprep kit, Zymo Research, Irvine, CA), 20 ng of total nucleic acid from each sample were enzymatically sheared with fragmentase (New England Biolabs, Ipswich, MA), and sequencing libraries were constructed using the NEBNext Ultra II Library Prep Kit (New England Biolabs). Samples underwent adaptor ligation and multiplexing with unique dual-indexing primers and then followed with polymerase chain reaction (PCR) amplification (15 cycles) and solid-phase reversible immobilization cleanup to retain 450–base pair fragments. Libraries were quantified, pooled, and then sequenced on an Illumina (San Diego, CA) NextSeq550.

After the demultiplexing was completed, we processed raw sequencing files through the CZ ID pipeline (https://czid.org/) to detect microbial species and strains (Illumina mNGS pipeline v8.3, San Francisco, CA) and ARGs (AMR pipeline v1.3.2, San Francisco, CA).^12^ The CZ AMR pipeline implements the Comprehensive Antibiotic Resistance Database (CARD) Resistance Gene Identifier tool^13^^,^^14^ which aligns quality-controlled reads against a continually updated, curated reference database of ARGs. We included ARGs specific to these antibiotic classes: aminoglycoside, β-lactam, chloramphenicol, diaminopyrimidine/sulfonamide, fluoroquinolone, fosfomycin, macrolide-lincosamide-streptogramin, nitroimidazole, peptide, and tetracycline. Only ARGs with 100% read coverage breadth were retained for analyses. We identified ESBL genes among *Enterobacterales* isolates.

We evaluated isolate genetic relatedness using the single–nucleotide polymorphism (SNP) Pipeline for Infectious Disease (SPID).^15^^,^^16^ SPID utilizes minimap2 to align short reads to a primary reference genome and call a consensus nucleotide at each position, enabling the calculation of SNP distance between all sample pairs.^17^ Maximum likelihood phylogenetic analyses were performed using RAxML-NG following Multiple Sequence Alignment methods, and phylogenetic trees were constructed using best tree fit.^18^ Isolates with <10 SNPs across >80% of the pathogen genome were considered closely related.

### Clinical data.

Using the hospital’s electronic and paper medical records and information from guardians, we extracted patient characteristics, medical interventions, diagnostic results, hospital course, and final hospital outcomes and entered them into a secure REDCap database.^19^ Severity of illness was characterized using the Lambaréné Organ Dysfunction (LOD) mortality prediction score.^20^

## STATISTICAL ANALYSES

We used univariate descriptive statistics to summarize cohort-level information and tested for associations between participant characteristics and mortality using *t*-tests or Wilcoxon rank-sum tests (continuous variables) and χ^2^ or Fisher’s exact tests (categorical variables). A *P*-value <0.05 was considered statistically significant. We fit a logistic regression model to test the association between BSI and mortality and adjusted for confounders based on their significance in the literature (age,^2^ HIV infection,^2^ malnutrition,^21^ immunization status^2^). We performed statistical analyses using Stata/MP 18.0 (StataCorp, College Station, TX), and generated figures using Prism for Mac v10.0 (GraphPad, https://www.graphpad.com) and RStudio (v2024.09.0 + 375).

## RESULTS

**Figure 1.**
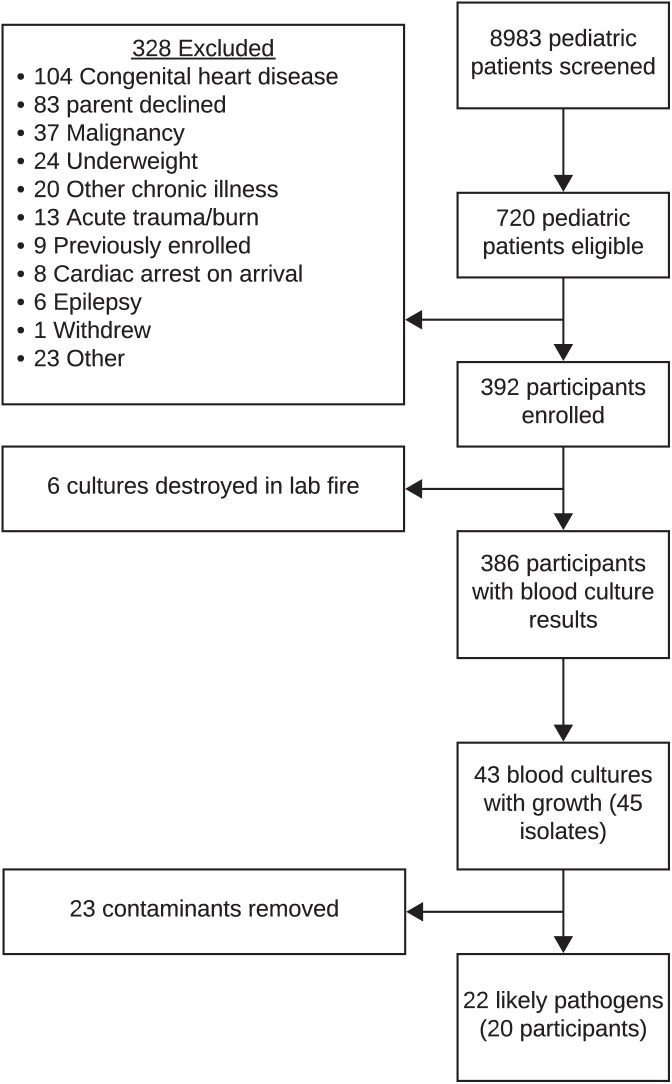
Flowchart depicting participant enrollment and blood culture isolates available for whole genome sequencing from Tanzanian children with severe febrile illness.

Of 8,983 children screened, 720 were eligible, and 392 were enrolled (Figure 1). Median age was 17.8 months (interquartile range [IQR] 9.1, 42.7), and 57.7% (*n* = 226) were male (Table 1). Among all participants, 30.8% (*n* = 121) had moderate–severe malnutrition, 1.8% (*n* = 7) were living with HIV, 4.6% (*n* = 18) tested positive for malaria, and 24.5% (*n* = 95) required pediatric intensive care unit (PICU) admission.

**Table 1:** Participant characteristics for children with severe febrile illness in Tanzania by survival status

Baseline Characteristic	Total (*N* = 392)	Survivors (*n =* 323)	Nonsurvivors (*n =* 69)	*P-*Value
Age (months), median (IQR)	17.8 (9.1, 42.7)	19.9 (10.3, 46.7)	11.4 (6.6, 23.9)	0.002
Male sex, *n* (%)	226 (57.7)	187 (57.9)	39 (56.5)	0.83
Moderate-severe malnutrition, *n* (%)	121 (30.9)	87 (26.9)	34 (49.3)	<0.001
HIV positive, *n* (%)	7 (1.8)	2 (0.6)	5 (7.2)	<0.001
Malaria positive, *n* (%)	18 (4.6)	15 (4.6)	3 (4.3)	0.92
Fully vaccinated, *n* (%)	389 (99.2)	321 (99.4)	68 (98.6)	0.47
LOD score, mean (SD)	1.1 (0.9)	0.9 (0.8)	2.1 (0.8)	<0.001
Hemoglobin g/dL, median (IQR)	9.6 (8.1, 11.1)	9.9 (8.5, 11.2)	8.6 (6.9, 10.1)	<0.001
White blood cell count per *µ*L, median (IQR)	10.3 (6.9, 15.1)	9.9 (6.6, 14.2)	13.9 (9.6, 19.0)	<0.001
Prehospital antibiotics, *n* (%)	181 (46.2)	134 (41.5)	47 (68.1)	<0.001
Bloodstream infection, *n* (%)	20 (5.2)	11 (3.4)	9 (13.4)	<0.001
Outcome				
PICU admission, *n* (%)	95 (24.5)	43 (13.5)	52 (75.4)	<0.001
Intubation, *n* (%)	62 (15.8)	21 (6.5)	41 (59.4)	<0.001

HIV = human immunodeficiency virus; IQR = interquartile range; LOD = Lambaréné organ dysfunction; PICU = pediatric intensive care unit; RDT = rapid diagnostic test; SD = standard deviation.

All–cause in-hospital mortality was 17.6% (*n* = 69). The prevalence of culture-confirmed BSI among those with available blood cultures (*n* = 386) was 5.2% (*n* = 20), and the case fatality rate was 45% (*n* = 9/20). Mortality was significantly associated with BSI (9/69 [13.4%] versus 11/69 [3.4%], *P*-value <0.001), mean LOD score [2.1 (SD = 0.8) versus 0.9 (SD = 0.8), *P*-value <0.001], and PICU admission (52/69 [75.4%] versus 43/69 [13.5%], *P*-value <0.001). The unadjusted and adjusted odds ratios for mortality in participants with BSI compared with those without was 4.4 (95% CI: 1.7, 11.0) and 3.9 (95% CI: 1.5, 10.1), respectively. Compared with surviving participants, those who died were significantly younger (11.4 months [IQR 6.6, 23.9] versus 19.9 months [10.3, 46.7], *P*-value = 0.002), had increased moderate–severe malnutrition (34 [49.3%] versus 87 [26.9%], *P*-value <0.001), and were more likely to be living with HIV (5 [7.2%] versus 2 [0.6%], *P*-value <0.001).

Before the EMD presentation, 46.2% (*n* = 181) of the participants received antibiotics, among whom 45.3% (*n* = 82/181) received ceftriaxone (Table 2). Empiric antibiotics were administered in the EMD to 63.3% (*n* = 248) of the participants, and it was primarily ceftriaxone (91.9%, *n* = 228/248) and metronidazole (40.3%, *n* = 100/248). The most common in-hospital antibiotics included ceftriaxone (72.5%, *n* = 177/244), metronidazole (13.1%, *n* = 32/244), and amoxicillin-clavulanate (13.1%, *n* = 32/244). The most common infectious diagnoses were acute watery diarrhea (26%, *n* = 101), sepsis (13.9%, *n* = 54), and pneumonia (11.8%, *n* = 46), whereas 20.8% (*n* = 81) of participants did not have an infection diagnosed (Supplemental Table 2).

**Table 2:** Antibiotic administration prehospital and empiric antibiotic administration in the Emergency Medicine Department for children with severe febrile illness in Tanzania (*N* = 392)

Antibiotic	*n* (%)		
	Prehospital	Empiric EMD	During Hospitalization
Any antibiotic received	181 (46.2)	248 (63.3)	244 (62.2)
Ceftriaxone	82 (20.9)	228 (58.2)	177 (45.2)
Metronidazole	31 (7.9)	100 (25.5)	32 (8.2)
Amoxicillin-clavulanate	27 (6.9)	13 (3.3)	32 (8.2)
Gentamicin	23 (5.9)	–	10 (2.6)
Ampicillin-cloxacillin	22 (5.6)	1 (0.3)	3 (0.8)
Ampicillin	8 (2)	–	5 (1.3)
Ciprofloxacin	7 (1.8)	1 (0.3)	20 (5.1)
Amoxicillin	6 (1.5)	1 (0.3)	–
Penicillin	5 (1.3)	–	6 (1.5)
Cotrimoxazole	4 (1.0)	–	10 (2.6)
Erythromycin	4 (1.0)	–	–
Azithromycin	4 (1)	–	19 (4.8)
Doxycycline	1 (0.3)	1 (0.3)	–
Meropenem	1 (0.3)	–	29 (7.4)
Piperacillin-tazobactam	–	–	13 (3.3)
Vancomycin	–	–	5 (1.3)
RIPE therapy	–	–	5 (1.3)
Amikacin	–	–	1 (0.3)
Cefepime	–	–	1 (0.3)
Chloramphenicol	–	–	1 (0.3)
Unknown	9 (2.3)	–	–
Other	6 (1.5)	–	15 (3.9)

EMD = Emergency Medicine Department; RIPE = rifampin, isoniazid, pyrazinamide, ethambutol.

**Figure 2.**
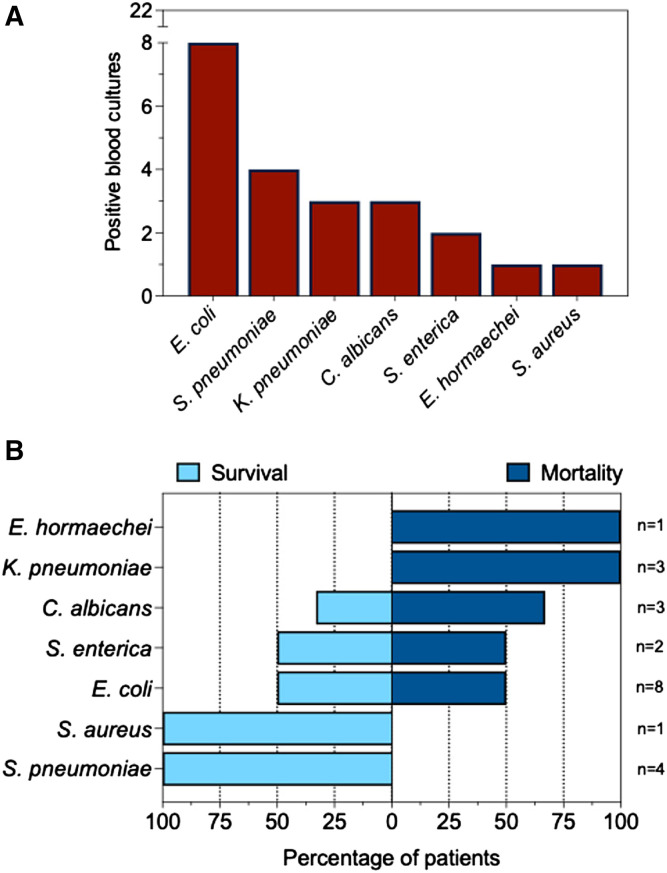
Bloodstream infection (BSI) pathogens detected and associations with mortality are presented. (**A**) Prevalence of BSI pathogens identified by culture. (**B**) Mortality associated with BSI pathogens.

Baseline characteristics and final diagnoses between participants with and without a BSI were similar (Supplemental Tables 3 and 4). Among the 20 positive blood cultures, 22 pathogens were identified as the following: 14 gram-negative bacteria (*Escherichia coli*, *n* = 8; *Klebsiella pneumoniae*, *n* = 3; *Salmonella enterica*, *n* = 2; *Enterobacter hormaechei*, *n* = 1), 5 gram-positive bacteria (*Streptococcus pneumoniae*, *n* = 4; *Staphylococcus aureus*, *n* = 1), and 3 cases of *Candida albicans* fungemia (Figure 2A). Mortality was highest in participants with gram-negative bacterial infections or fungemia (Figure 2B).

**Figure 3.**
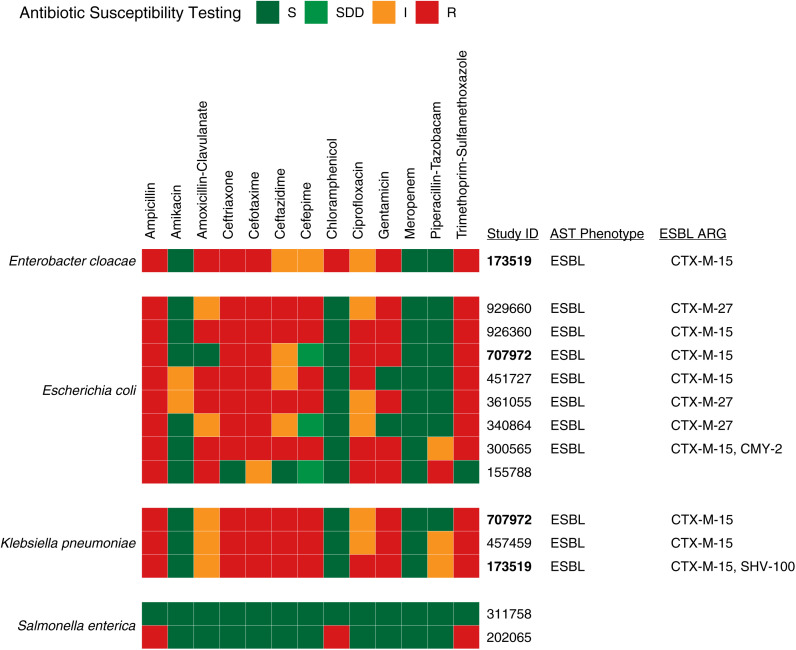
Antimicrobial resistance genes detected in gram-negative blood culture isolates from Tanzanian children with severe febrile illness. Phenotypic antimicrobial sensitivity testing (AST) results and corresponding whole genome sequencing-detected antimicrobial resistance genes (ARGs) of the *Enterobacterales* spp. isolates. Isolates are identified by a 6-digit study identification (ID) number. Participants: Study ID numbers bolded in black indicate participants with blood cultures that grew two different bacterial isolates. ESBL = extended–spectrum β-lactamase producers; I = intermediate; R = resistant; S = Sensitive; SDD = sensitivity dose-dependent.

**Figure 4.**
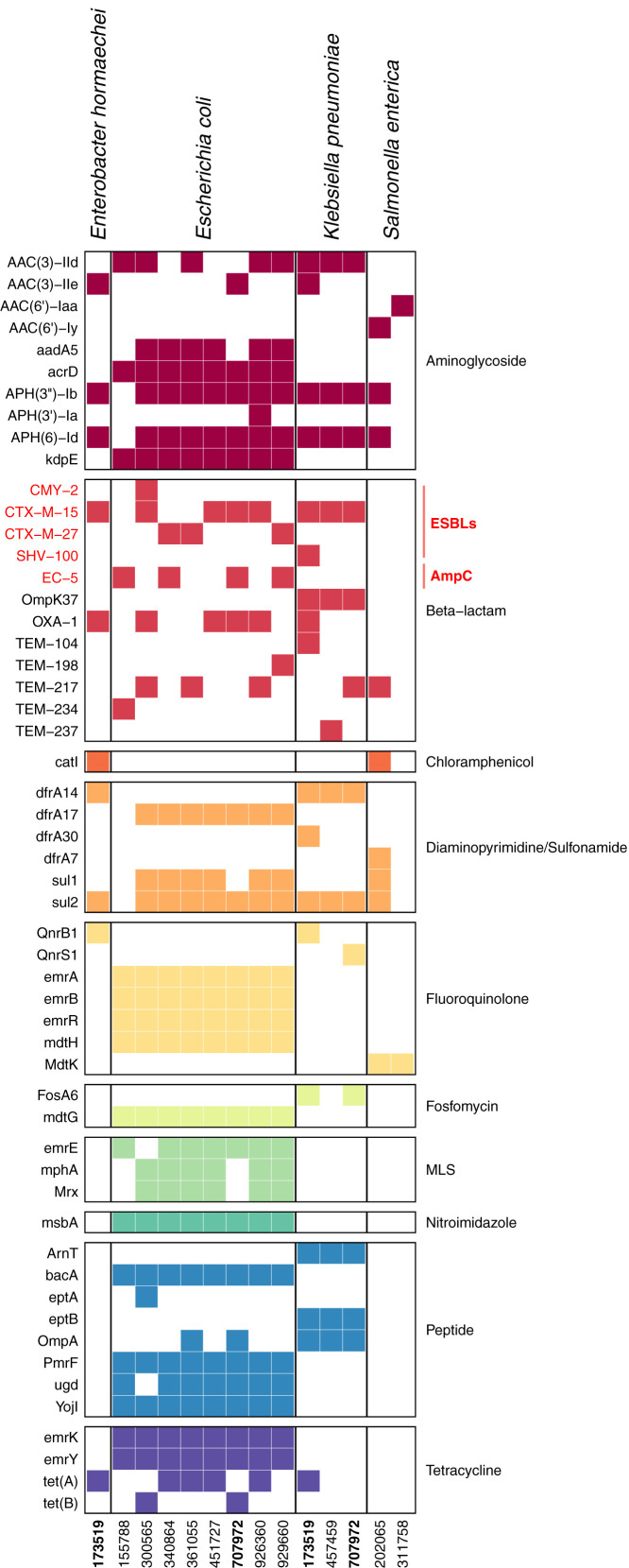
Antimicrobial resistance genes (ARGs) in gram-negative bloodstream pathogens identified in children with severe febrile illness. Isolates are identified by a 6-digit study identification (ID) number. ARGs, grouped by class, were identified from whole genome sequencing of gram-negative bacterial pathogens detected by blood culture. Extended–spectrum β-lactamase and *AmpC*-like β-lactam resistance genes conferring ceftriaxone resistance are highlighted in red. Study IDs bolded black indicate participants with blood cultures that grew two different bacterial isolates.

Eleven (79%) of 14 gram-negative bacterial pathogens displayed phenotypic antimicrobial resistance to ceftriaxone or ceftazidime, suggesting ESBL production (Figure 3). These 11 isolates harbored *CTX-M* by WGS; 8 isolates had *CTX-M-15*, and 3 isolates had *CTX-M-27* (Figures 3 and 4). Interestingly, the most common ESBL producers were *E. coli* (7 of 8 isolates, 87.5%) and *K. pneumoniae* (3 of 3 isolates, 100%). The single *E. coli* isolate without an ESBL–producing phenotype had an *Amp-C–*like β-lactamase gene (*EC-5*), which can induce resistance to higher-generation cephalosporins in the presence of antibiotics (Figure 4). Isolates from the same bacterial species shared similar ARG profiles, with the greatest intraspecies variation in the β-lactam ARG class (Figure 4). A comparatively smaller number of AMR genes was identified in gram-positive BSI pathogens. The *S. aureus* isolate was methicillin–resistant, and three of the four *S. pneumoniae* isolates were penicillin–resistant. All gram-positive isolates were susceptible to clindamycin (Supplemental Figure 1). Fewer ARGs were identified in gram-positive (median [IQR]: 4 [4–4] ARGs) compared with gram-negative pathogens (median [IQR]: 21 [11–26] ARGs) (Figure 4 and Supplemental Figure 2).

Finally, we evaluated the genetic relatedness of bacterial and fungal blood culture isolates. None of the *E. coli* or *K. pnuemoniae* BSIs involved genetically related isolates (Supplemental Figures 3 and 4). However, two of four patients with *S. pneumoniae* BSIs (Supplemental Figure 5) and two of three participants with candidemia had genetically identical isolates (Supplemental Figure 6).

## DISCUSSION

In a prospective cohort of 392 Tanzanian children with SFI, we found that culture-confirmed BSI is associated with a fourfold increase in odds of mortality. Furthermore, we reported that the majority of identified BSI pathogens are resistant to ceftriaxone, the first-line antimicrobial regimen recommended by Tanzanian National Guidelines for the treatment of SFI.^22^ Using WGS, we further demonstrated that the majority of gram-negative pathogens harbored *CTX-M* or other ESBL genes and uncovered evidence of potential *S. pneumoniae and Candida* pathogen transmission in the community.

Mortality in children with BSI was 45%, notably higher than the 8% mortality observed in a previous cohort of young children with acute febrile illness hospitalized in four Tanzanian public hospitals, including MNH.^3^ Mortality in our cohort was also higher than that in a study of 19 sites primarily in LMICs, which reported 18% mortality in infants hospitalized with sepsis due to a pathogen-positive culture.^23^ These differences may be due to delayed presentation and/or increased severity of illness.^24^ However, the prevalence of BSI (5%) in our cohort was lower than that reported in a previous Tanzanian pediatric acute febrile illness cohort (11%) or in a global observational study of infants with sepsis (18%).^3^^,^^23^ Because our study site was a national referral hospital, participants were frequently evaluated at other facilities before presentation, which presumably contributed to the high rate of prehospital antibiotic use (46%), which may have rendered cultures falsely negative.

With the use of locally available clinical diagnostics, a definitive BSI pathogen (bacterial, fungal, or parasite) was identified in only 10% of cohort participants. In a systematic review of SFI in East Africa (29,286 adults and children) that included studies incorporating more comprehensive diagnostics (e.g., PCR, serology), pathogens were identified in 18% of participants.^5^ Together, this illustrates that the underlying infectious etiology of SFI is not identified in most cases, and there is an urgent need for more advanced, culture-independent diagnostic approaches such as metagenomic sequencing to improve our understanding of SFI etiologies in this population.

Importantly, phenotypic and genotypic AMR analyses among identified pathogens demonstrated a high prevalence of *Enterobacterales* resistant to ceftriaxone, the first-line empiric antibiotic for pediatric febrile illness at MNH. Ceftriaxone, which is widely available and inexpensive, is used throughout SSA for the empiric management of pediatric infections,^8^^,^^25^ consistent with both local and national guidelines.^22^ Ceftriaxone’s primary coverage gaps include ESBL and *AmpC–*producing gram-negative organisms, anaerobes, and some gram-positive pathogens such as *Staphylococcus* or *Enterococcus* spp.

Among the BSI pathogens identified, ceftriaxone would normally be considered the proper treatment for 18/22 pathogens detected (excluding *S. aureus* bacteremia or fungemia), and its empiric use would have been effective. However, because of the high prevalence of ESBL production among *Enterobacterales* pathogens, only seven isolates (4/4 *S. pneumoniae*, 2/2 *S. enterica*, and 1/8 *E. coli*) were susceptible to ceftriaxone. Furthermore, all ESBL–producing blood pathogens had *CTX-M*, the most common ESBL gene type worldwide.^26^ This aligns with findings from other SSA pediatric AMR studies that demonstrate a high prevalence of ESBL–producing organisms,^6^ including a recent cross-sectional study of young children in Tanzania, where >50% of hospitalized children with bacteremia were infected with ESBL and/or multidrug-resistant organisms.^3^

In this cohort of children with community-acquired BSI, we observed multiple genetically unrelated ESBL *E. coli* and *K. pneumoniae* isolates. The lack of genetic relatedness between these isolates reinforces the significance and pervasiveness of AMR in the community. Concerningly, multiple studies demonstrate an increased prevalence of ESBL–producing organisms *S. pneumoniae* and *C. albicans* circulating in the community and causing infections.^8^^,^^27^^–^^30^ Our findings of genetically related *S. pneumoniae* and *C. albicans* infections highlight the importance of community-based transmission.

This study has several strengths. It is a prospective pediatric cohort in SSA where the etiology of SFI is not well understood, it includes detailed clinical phenotyping, and it incorporates WGS. It also has limitations, including a reliance on routine blood culture, which can have poor sensitivity for detecting pathogens in the blood, particularly if patients are pretreated with antibiotics or inadequate blood volumes are obtained. Although microbial culture is the gold standard, culture-independent techniques (e.g., metagenomic sequencing) may have identified additional missed infections, including those pretreated with antibiotics. Second, the study site was a tertiary care referral hospital that cares for the most severe cases. This cohort may not be representative of lower-level hospital populations or of the community. Third, the relatively small number of bacterial and fungal pathogens isolated in the cultures limited our capacity to thoroughly evaluate pathogen-specific associations with mortality.

In this pediatric SFI cohort, BSI conferred high mortality and was frequently caused by ESBL–producing gram-negative organisms resistant to first-line antibiotics. Additional surveillance studies across SSA are needed to confirm these findings and guide revision of empiric antimicrobial regimens. Reducing the burden of pediatric SFI and AMR requires a multifaceted approach, including prevention, evidence-based empiric antimicrobial use, improved access to timely, accurate diagnostics, and AMR surveillance.

## Supplemental Materials

10.4269/ajtmh.25-0522 Supplemental Materials

## Data Availability

Raw sequencing reads are available from the National Center for Biotechnology Information Sequence Read Archive accession SUB14909672.
