# Supplementary material for: Prospective Genomic Surveillance of Severe Febrile Illness in Tanzanian Children Identifies High Mortality and Resistance to First-Line Antibiotics in Bloodstream Infections
Source: Am J Trop Med Hyg. 2026 Feb 26;114(4):767–73. doi: 10.4269/ajtmh.25-0522 (PMC13045537; doi:10.4269/ajtmh.25-0522)

SUPPLEMENTAL MATERIALS

SUPPLEMENTAL TABLES

Supplemental Table 1. Operational definitions for World Health Organization warning signs

**Warning Sign**	**Definition**
Respiratory Distress	Presence of **one or more** of the following:<br>• Tachypnea for age<br>• Increased work of breathing (presence of retractions, flaring or head bobbing)<br>• Hypoxia (SaO2 <92%) OR on oxygen
Altered Mental Status	Presence of **one or more** of the following:<br>• A score of ‘Verbal’, ‘Pain’ or ‘Unresponsive’ on the Alert-Verbal-Pain-Unresponsive (AVPU) scale<br>• History of convulsion within the last 24 hours in a patient without known epilepsy
Impaired Perfusion	Presence of **one or more** of the following:<br>• Hypotension for age<br>• Cold extremities<br>• Delayed capillary refill<br>• Severe tachycardia for age
At-risk for dehydration	Presence of **one or more** of the following:<br>• ≥5 loose stools over the past 24 hours<br>• ≥3 loose stools over past 24 hours and emesis<br>• ≥3 emesis over past 24 hours<br>• Decreased urine output per guardian report; or inability to drink or breastfeed

Supplemental Table 2. Final infectious diagnosis at time of hospital discharge or death for children with severe febrile illness in Tanzania by survival status[a]

Diagnosis	Total (n=392)	Survivors (n=323)	Non-Survivors (n=69)	p value
Acute watery diarrhea	101 (26.0)	99 (30.7)	2 (2.9)	<0.001
Bronchiolitis	7 (1.8)	7 (2.2)	0 (0.0)	0.22
Fever of unknown origin	1 (0.3)	1 (0.3)	0 (0.0)	0.64
HIV/AIDs	10 (2.6)	2 (0.6)	8 (11.6)	<0.001
Malaria	15 (3.9)	13 (4.0)	2 (2.9)	0.66
Measles	11 (2.8)	11 (3.4)	0 (0.0)	0.12
Meningitis/Encephalitis	25 (6.4)	19 (5.9)	6 (8.7)	0.39
Pneumonia	46 (11.8)	37 (11.4)	9 (13.0)	0.60
Sepsis	54 (13.9)	25 (7.7)	29 (42.0)	<0.001
Skin and soft tissue infection	3 (0.8)	3 (0.9)	0 (0.0)	0.42
Tuberculosis	4 (1.0)	1 (0.3)	3 (4.3)	0.002
Upper respiratory tract infection	20 (5.1)	20 (6.2)	0 (0.0)	0.03
Urinary tract infection	11 (2.8)	11 (3.4)	0 (0.0)	0.12
No infection diagnosed	81 (20.8)	71 (22.0)	10 (14.5)	0.16

[a] HIV, Human Immunodeficiency Virus; AIDs, Acquired Immunodeficiency Syndrome

Supplemental Table 3. Participant baseline characteristics and outcomes for children with severe febrile illness in Tanzania by bloodstream infection (BSI) status; limited to those with blood culture results (n=386) [a]

Baseline Characteristic	Total (n=386)	No BSI (n=366)	Bloodstream infection (n=20)	p value
Age (months), median (IQR)	17.8 (9.1, 42.4)	18.2 (9.4, 42.4)	11.9 (7.8, 42.4)	0.24
Male sex, n (%)	222 (57.5)	210 (57.4)	12 (60.0)	0.82
Moderate-Severe malnutrition, n (%)	119 (30.8)	110 (30.1)	9 (45.0)	0.16
HIV positive, n (%)	7 (1.8)	6 (1.6)	1 (5.0)	0.27
Malaria status by RDT, n (%)	18 (4.7)	18 (4.9)	0 (0.0)	0.31
Immunization status, n (%)	384 (99.5)	364 (99.5)	20 (100.0)	0.74
LOD score, mean (SD)	1.1 (0.9)	1.1 (0.9)	1.3 (1.0)	0.32
Hemoglobin g/dL, median (IQR)	9.6 (8.2, 11.1)	9.7 (8.2, 11.1)	8.6 (6.8, 10.1)	0.023
White blood cell count per uL, median (IQR)	10.3 (6.9, 15.0)	10.2 (6.9, 14.8)	15.1 (6.3, 20.3)	0.18
Pre-Hospital antibiotics, n (%)	177 (45.9)	165 (45.1)	12 (60.0)	0.19
Outcome				
In hospital mortality, n (%)	67 (17.4)	58 (15.8)	9 (45.0)	<0.001
PICU admission, n (%)	93 (24.3)	86 (23.7)	7 (36.8)	0.19
Intubation, n (%)	59 (15.3)	56 (15.3)	3 (15.0)	0.97

[a] HIV, Human Immunodeficiency Virus; LOD, Lambaréné Organ Dysfunction; IQR, Interquartile Range; RDT, Rapid Diagnostic Test; SD, Standard Deviation; uL, microliter; g, gram; dL, deciliter; PICU, Pediatric Intensive Care Unit

Supplemental Table 4. Final infectious diagnosis at time of hospital discharge or death for children with severe febrile illness in Tanzania by bloodstream infection (BSI) status; limited to those with blood culture results (n=386) [a]

Diagnosis	Total (n=386)	No BSI (N=366)	BSI (N=20)	p value
Acute watery diarrhea	98 (25.6)	94 (25.8)	4 (21.1)	0.57
Bronchiolitis	7 (1.8)	7 (1.9)	0 (0.0)	0.53
Fever of unknown origin	1 (0.3)	1 (0.3)	0 (0.0)	0.81
HIV/AIDs	10 (2.6)	9 (2.5)	1 (5.3)	0.49
Malaria	15 (3.9)	15 (4.1)	0 (0.0)	0.36
Measles	11 (2.9)	11 (3.0)	0 (0.0)	0.43
Meningitis/Encephalitis	24 (6.3)	24 (6.6)	0 (0.0)	0.24
Pneumonia	44 (11.5)	46 (12.6)	0 (0.0)	0.099
Sepsis	52 (13.6)	44 (12.1)	8 (42.1)	<0.001
Skin and soft tissue infection	3 (0.8)	3 (0.8)	0 (0.0)	0.68
Tuberculosis	4 (1.0)	4 (1.1)	0 (0.0)	0.64
Upper respiratory tract infection	20 (5.2)	19 (5.2)	1 (5.3)	0.97
Urinary tract infection	11 (2.9)	10 (2.7)	1 (5.3)	0.55
No infection diagnosed	81 (21.1)	77 (21.2)	4 (21.1)	0.91

[a] HIV, Human Immunodeficiency Virus; AIDs, Acquired Immunodeficiency Syndrome

SUPPLEMENTAL FIGURES

Supplemental Figure 1. Antimicrobial resistance genes (ARGs) detected in gram-positive bacterial blood culture isolates from Tanzanian children with severe febrile illness. Phenotypic antimicrobial sensitivity testing results and corresponding whole genome sequencing-detected antimicrobial resistance genes. Isolates identified by 6-digit study ID. Participants; Study IDs bolded black indicate participants with blood cultures that grew two different bacterial isolates. S: Sensitive; SDD: Sensitivity Dose-Dependent; I: Intermediate; R: Resistant.

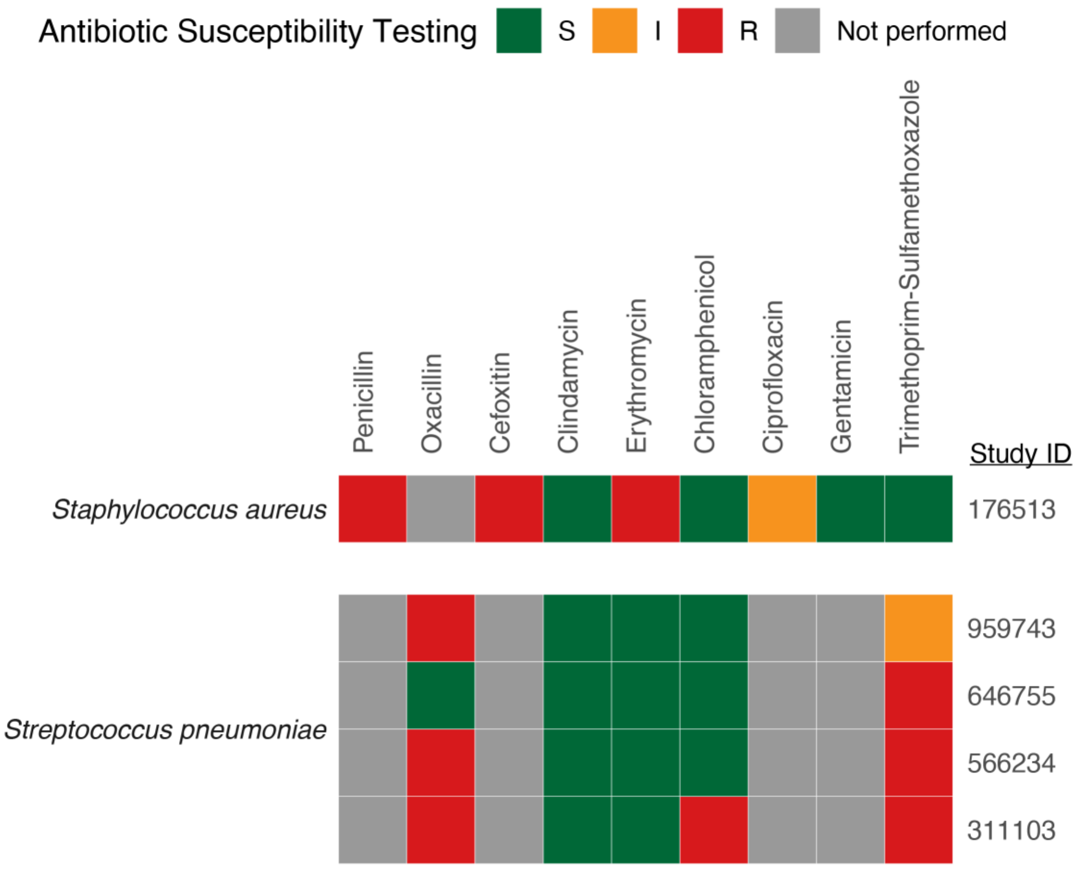

Supplemental Figure 2. Antimicrobial resistance genes (ARGs) in gram-positive bloodstream pathogens identified in children with severe febrile illness. Isolates identified by 6-digit study ID. ARGs, grouped by class, identified from WGS of gram-negative bacterial pathogens detected by blood culture. Study IDs bolded black indicate participants with blood cultures that grew two different bacterial isolates**.**

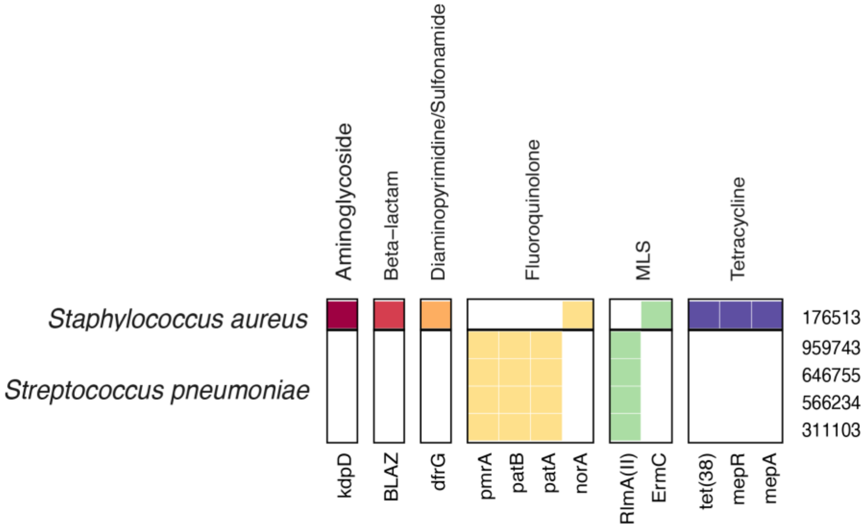

Supplemental Figure 3. Maximum likelihood phylogenetic tree based on *E. coli* genomes recovered from blood culture isolates. Red = subject IDs. Grey = reference genomes. Scale bar indicates substitutions per site.

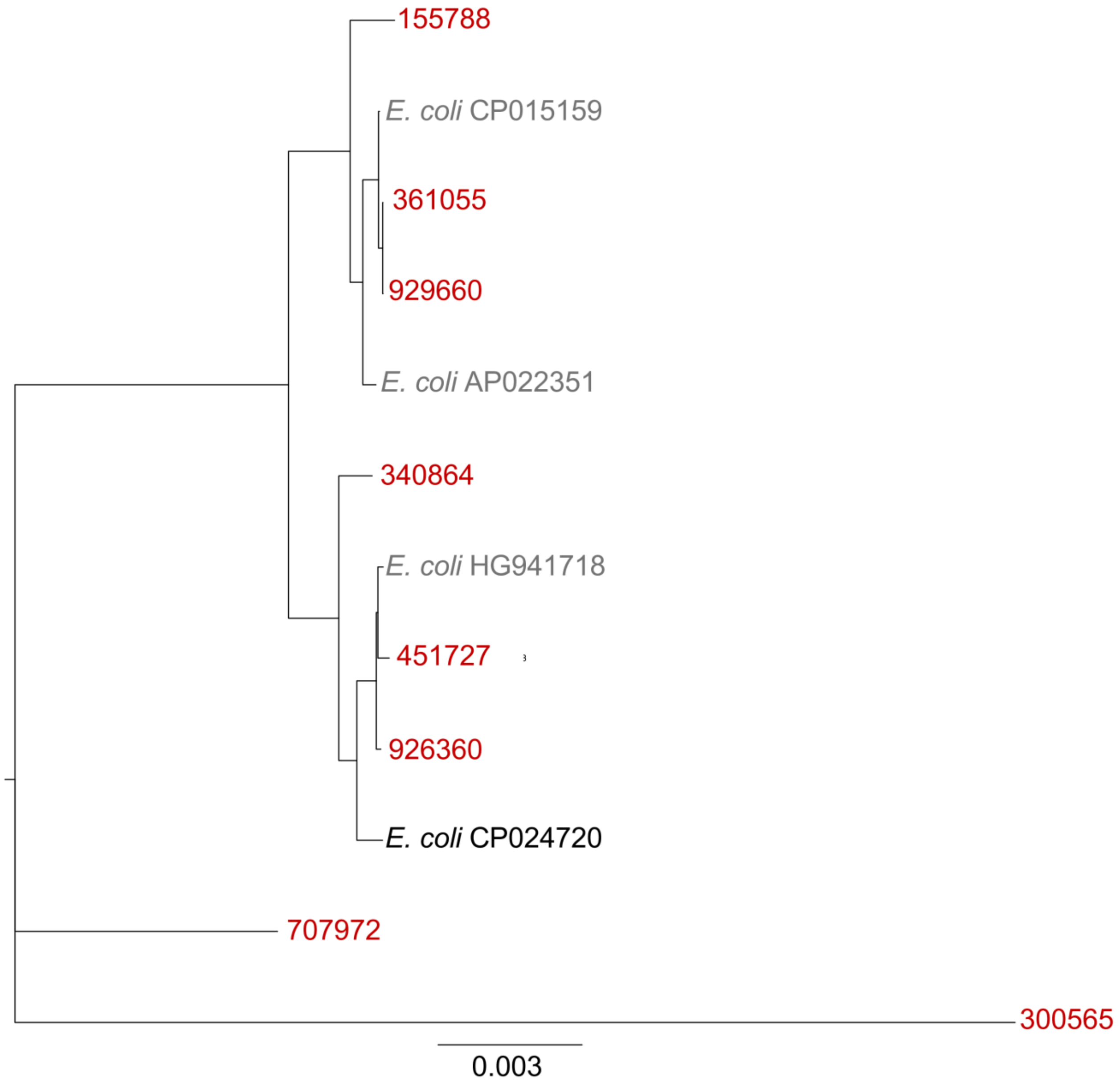

Supplemental Figure 4. Maximum likelihood phylogenetic tree based on *K. pneumoniae* genomes recovered from blood culture isolates. Red = subject IDs. Grey = reference genomes. Scale bar indicates substitutions per site.

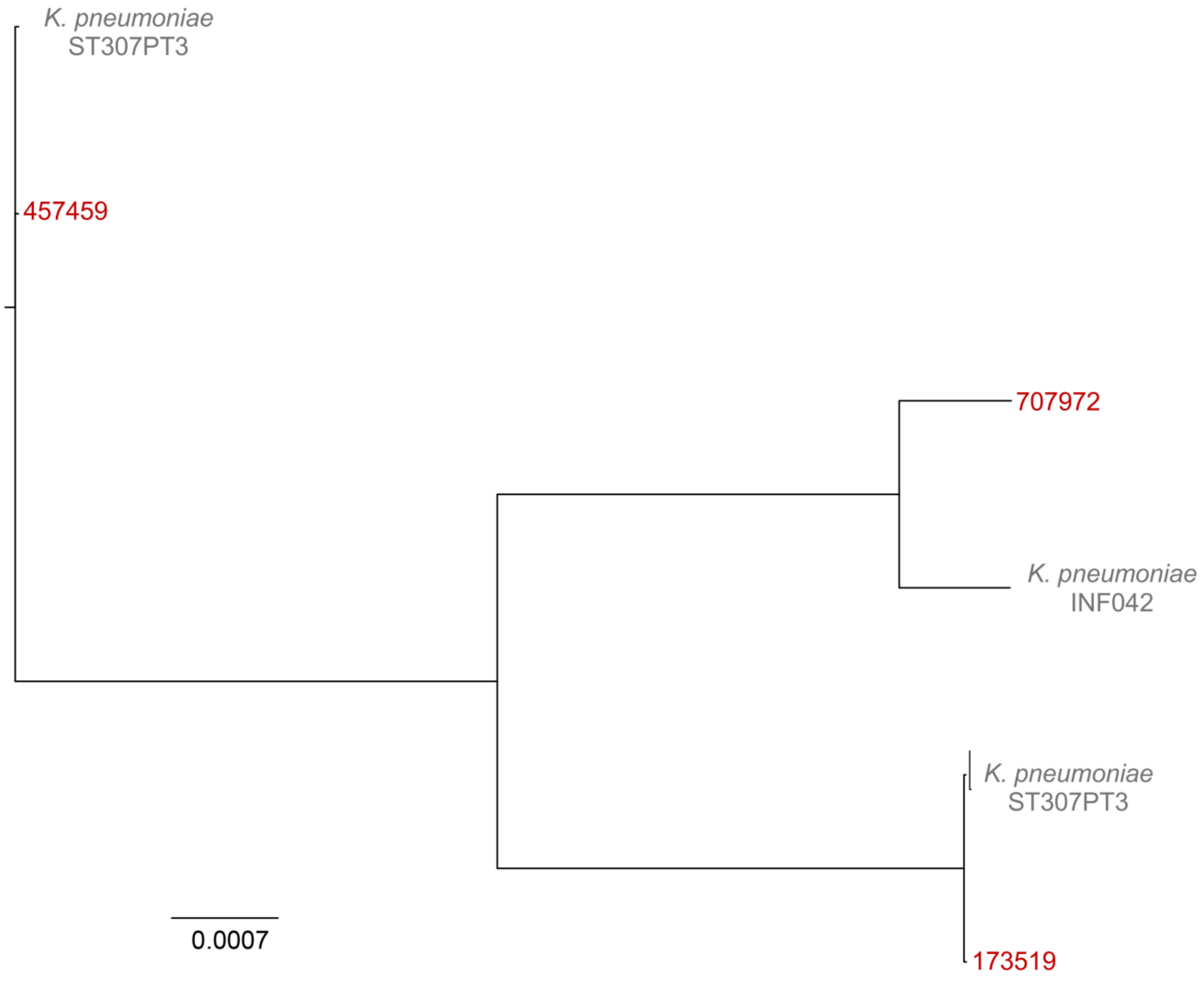

Supplemental Figure 5. Maximum likelihood phylogenetic tree based on *S. pneumoniae* genomes recovered from blood culture isolates. Red = subject IDs. Grey = reference genomes. Scale bar indicates substitutions per site.

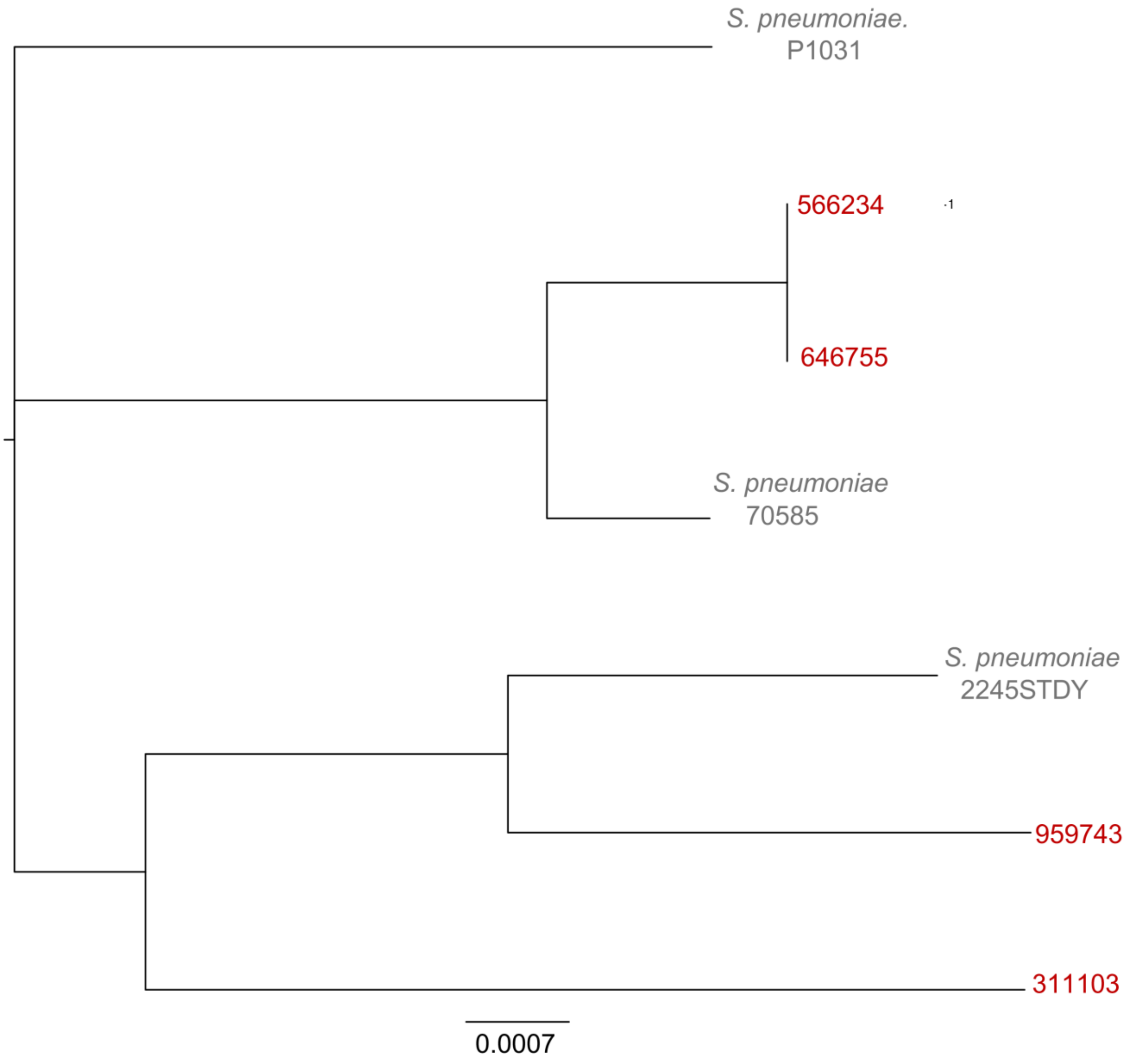

Supplemental Figure 6. Maximum likelihood phylogenetic tree based on *C. albicans* genomes recovered from blood culture isolates. Red = subject IDs. Grey = reference genomes. Scale bar indicates substitutions per site.

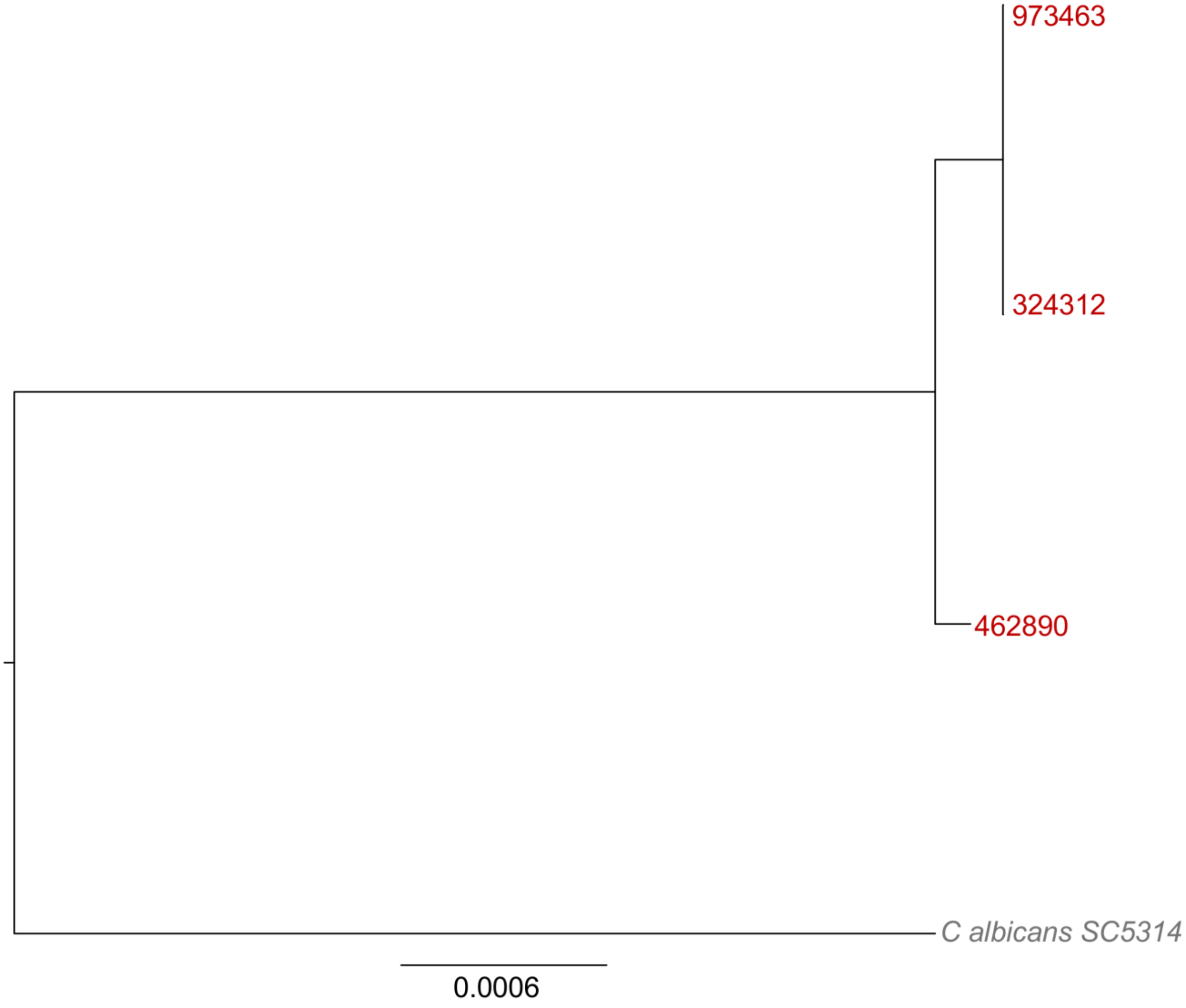

Supplement: Supplemental Materials [file tpmd250522.SD1.pdf]
